# Motor Deficits in the Ipsilesional Arm of Severely Paretic Stroke Survivors Correlate With Functional Independence in Left, but Not Right Hemisphere Damage

**DOI:** 10.3389/fnhum.2020.599220

**Published:** 2020-12-09

**Authors:** Shanie A. L. Jayasinghe, David Good, David A. Wagstaff, Carolee Winstein, Robert L. Sainburg

**Affiliations:** ^1^Department of Neurology, Pennsylvania State University College of Medicine, Hershey, PA, United States; ^2^Department of Human Development and Family Studies, Pennsylvania State University, State College, PA, United States; ^3^Department of Biokinesiology and Physical Therapy, University of Southern California, Los Angeles, CA, United States; ^4^Department of Kinesiology, Pennsylvania State University, State College, PA, United States

**Keywords:** CVA, motor deficits, functional outcome, kinematics, upper extremity

## Abstract

Chronic stroke survivors with severe contralesional arm paresis face numerous challenges to performing activities of daily living, which largely rely on the use of the less-affected ipsilesional arm. While use of the ipsilesional arm is often encouraged as a compensatory strategy in rehabilitation, substantial evidence indicates that motor control deficits in this arm can be functionally limiting, suggesting a role for remediation of this arm. Previous research has indicated that the nature of ipsilesional motor control deficits vary with hemisphere of damage and with the severity of contralesional paresis. Thus, in order to design rehabilitation that accounts for these deficits in promoting function, it is critical to understand the relative contributions of both ipsilesional and contralesional arm motor deficits to functional independence in stroke survivors with severe contralesional paresis. We now examine motor deficits in each arm of severely paretic chronic stroke survivors with unilateral damage (10 left-, 10 right-hemisphere damaged individuals) to determine whether hemisphere-dependent deficits are correlated with functional independence. Clinical evaluation of *contralesional, paretic* arm impairment was conducted with the upper extremity portion of the Fugl-Meyer assessment (UEFM). *Ipsilesional arm* motor performance was evaluated using the Jebsen-Taylor Hand Function Test (JTHFT), grip strength, and ipsilesional high-resolution kinematic analysis during a visually targeted reaching task. Functional independence was measured with the Barthel Index. Functional independence was better correlated with ipsilesional than contralesional arm motor performance in the left hemisphere damage group [JTHFT: [*r*_(10)_ = −0.73, *p* = 0.017]; grip strength: [*r*_(10)_ = 0.64, *p* = 0.047]], and by contralesional arm impairment in the right hemisphere damage group [UEFM: [*r*_(10)_ = 0.66, *p* = 0.040]]. Ipsilesional arm kinematics were correlated with functional independence in the left hemisphere damage group only. Examination of hemisphere-dependent motor correlates of functional independence showed that ipsilesional arm deficits were important in determining functional outcomes in individuals with left hemisphere damage only, suggesting that functional independence in right hemisphere damaged participants was affected by other factors.

## Introduction

The majority of stroke survivors have upper limb motor deficits (Kwakkel et al., 1999; Langhorne et al., 2009) that affect their functional independence and lead to a loss in overall quality of life. Physical rehabilitation research and clinical intervention in stroke tends to focus heavily on contralesional arm deficits because research has shown that impairment level is largely dependent on the extent and quality of movements in the paretic arm (de Niet et al., 2007; Lang et al., 2007). However, severely impaired stroke survivors, with little to no voluntary movement of the fingers, must rely on their ipsilesional, less-affected arm as a compensatory strategy in order to perform activities of daily living that require manipulation, thus placing a large functional burden on that arm.

Unfortunately, the ipsilesional arm of individuals with severe contralesional paresis tends to have substantial deficits in motor control and coordination that have been well elaborated in the literature (Jones et al., 1989; Winstein and Pohl, 1995; Goble and Brown, 2008; Barry et al., 2020). Importantly, motor deficits in the ipsilesional arm of stroke survivors depend on the hemisphere that was damaged (Schaefer et al., 2007; Sainburg, 2014, 2016), and have been predicted by our bi-hemispheric model of motor control that attributes predictive control of trajectory and limb dynamics to the left hemisphere, and control of limb impedance to the right hemisphere (Sainburg, 2002, 2016). Indeed, previous research with right-handed unilateral stroke survivors, using their ipsilesional arm, showed that left-hemisphere damaged (LHD) survivors tend to produce larger deficits in feedforward mechanisms that appear to specify trajectory direction and curvature (most apparent in the early stages of movement), as well as coordination with non-muscular forces, such as intersegmental inertial interactions (Haaland and Harrington, 1989b; Winstein and Pohl, 1995; Haaland et al., 2004; Schaefer et al., 2009a,b; Stewart et al., 2014; Sainburg, 2016; Varghese and Winstein, 2020). In contrast, right-hemisphere damaged (RHD) survivors produce larger deficits in impedance control mechanisms that are required to achieve accurate and stable postures at the end of movement, and to stabilize against unexpected mechanical perturbations. Due to the presence and lateralization of ipsilesional motor deficits, we expect that functional independence may be affected not only by the extent of paresis, but also by the deficits that are specific to each hemisphere.

Previous research has shown that ipsilesional motor deficits scale with the severity of contralesional arm impairment. A recent study that examined stroke survivors with mild-to-severe impairment found that the more severe the paresis, the greater the ipsilesional motor deficits. In addition, LHD individuals produced more severe deficits in ipsilesional arm functional performance than RHD individuals (Maenza et al., 2020). Although there is substantial evidence for hemispheric differences in motor control in both arms of stroke survivors with unilateral brain damage (Haaland and Harrington, 1989a,b; Winstein and Pohl, 1995; Mani et al., 2013), hemisphere-dependent differences in functional performance are less well-documented. In fact, in a previous study, we found that hemisphere-dependent differences in functional performance, measured using the Jebsen-Taylor Hand Function Test (JTHFT), were largely dependent on two processes, writing and simulated feeding, both of which are highly lateralized skills in typical adults (Maenza et al., 2020). Nevertheless, in another previous study, we showed that performance on that same functional performance measure (the JTHFT) was not significantly different between LHD and RHD individuals even though the underlying motor control deficits were different (Schaefer et al., 2009b). One factor that might give rise to the reported inconsistencies in ipsilesional arm impairment is that many studies examined motor deficits in individuals with large variation in contralesional paresis, which makes it difficult to determine whether the absence of hemisphere-dependent findings might arise from differences in severity of impairment between LHD and RHD groups. This is especially important, given our recent findings that ipsilesional arm motor deficits depend on the severity of contralesional arm impairment (Maenza et al., 2020).

The promotion of functional independence through physical rehabilitation in stroke survivors should benefit from understanding the potential hemisphere-specific correlates of functional independence in left- vs. right-hemisphere damaged survivors. To address this goal, we now examine the presence of hemisphere-dependent differences in motor control and functional performance, as reflected by high-resolution kinematic measures during reaching movements as well as lower-resolution clinical measures. We seek to determine whether these performance measures are correlated with functional independence, as measured by the Barthel Index (Mahoney and Barthel, 1965), a standard measure of functional independence. We restricted our recruitment to individuals with severe paresis because functional independence relies almost exclusively on performance of the ipsilesional arm, which is most affected in individuals with severe contralesional paresis (Maenza et al., 2020). We also restricted our recruitment to stroke survivors with self-reported premorbid right-handedness because previous findings of hemispheric specialization and hemisphere-specific motor deficits in stroke survivors have been restricted to right-handers, and little is known about hemisphere-specific motor deficits in left-handers. This gap in our knowledge is partially due to the low incidence of left-handers in the stroke population, but also because left-handers do not reflect a homogenous behavioral or neurologic population with regard to neural and motor lateralization (Hardyck and Petrinovich, 1977; Perelle and Ehrman, 1982).

We base our main hypothesis on our model of hemispheric specialization for motor control, which attributes predictive control of trajectory to the left hemisphere, and impedance-mediated control of steady-state position and motor corrections to the right hemisphere. We thus predict: (1) the presence of differential deficits in the initial and final accuracy of movements that depend on the lesioned hemisphere, (2) measures of functional independence will depend on ipsilesional, not contralesional arm, motor performance in both LHD and RHD survivors, given the severe limitations of the contralesional arm and the dependence on the ipsilesional arm for performance of activities of daily living, and (3) functional independence should be differentially determined by early trajectory deficits in LHD survivors and final position deficits in RHD survivors.

## Materials and Methods

### Participants

This study examined a convenience sample of first visit data from a larger, ongoing clinical intervention study that began in 2019. The present study reports data collected from 20 right-handed, chronic hemiparetic stroke survivors (10 LHD; 10 RHD; seven females; age 58 ± 10 years) with severe contralesional arm deficits, defined in previous work as less than 20 points on the upper-extremity portion of the Fugl-Meyer assessment (Michaelsen et al., 2001; Woodbury et al., 2013). A cluster analysis on the Fugl-Meyer assessment revealed that a score of less than 29 points could be classified as severe impairment (Woytowicz et al., 2017); however, this work showed that there was considerable overlap between the moderate and severe clusters, which we aimed to avoid by choosing a lower cutoff i.e., less than 20 points. We had an additional requirement of no voluntary finger movement in our participants, but mass finger flexion due to spasticity was allowed. This stringent focus on severely paretic stroke survivors had a substantial impact on recruitment, but we gained the ability to focus our analysis on this important group of stroke survivors, who are often overlooked in mechanistic and/or intervention research studies. Exclusion criteria were bilateral lesions, cognitive disability (determined as any major lack of attention or understanding of instructions), and non-stroke neurological diseases. The Edinburgh Handedness Inventory was provided to confirm hand dominance (Oldfield, 1971). We received written informed consent from all participants prior to study initiation. All study procedures were approved by either the Pennsylvania State University's or the University of Southern California's Institutional Review Boards (approval number: 8385).

### Experimental Design

#### Clinical Tests

We used two clinical tests to determine unilateral motor performance of the ipsilesional arm: (1) the Jebsen-Taylor hand function test (JTHFT) and (2) grip strength. The JTHFT consists of a series of timed hand coordination and manipulation tasks representative of activities of daily living (Jebsen et al., 1969). These tasks include writing, simulated page turning, stacking checkers, simulated feeding, and lifting objects of various sizes. Average grip strength (in kg) was calculated from three trials using a hand dynamometer (Lafayette instrument). We used the upper-extremity portion of the Fugl-Meyer assessment (UEFM) to assess contralesional arm impairment. The UEFM evaluates motor performance by examining reflex activity, movement synergies, passive and active movement (Fugl-Meyer et al., 1975).

We evaluated functional independence with the Barthel Index (BI, out of 100). The BI is a 10-item patient-reported outcome questionnaire that evaluates functional independence in activities of daily living, such as eating, dressing, grooming, toileting, and mobility.

#### Kinematics

**Figure 1:**
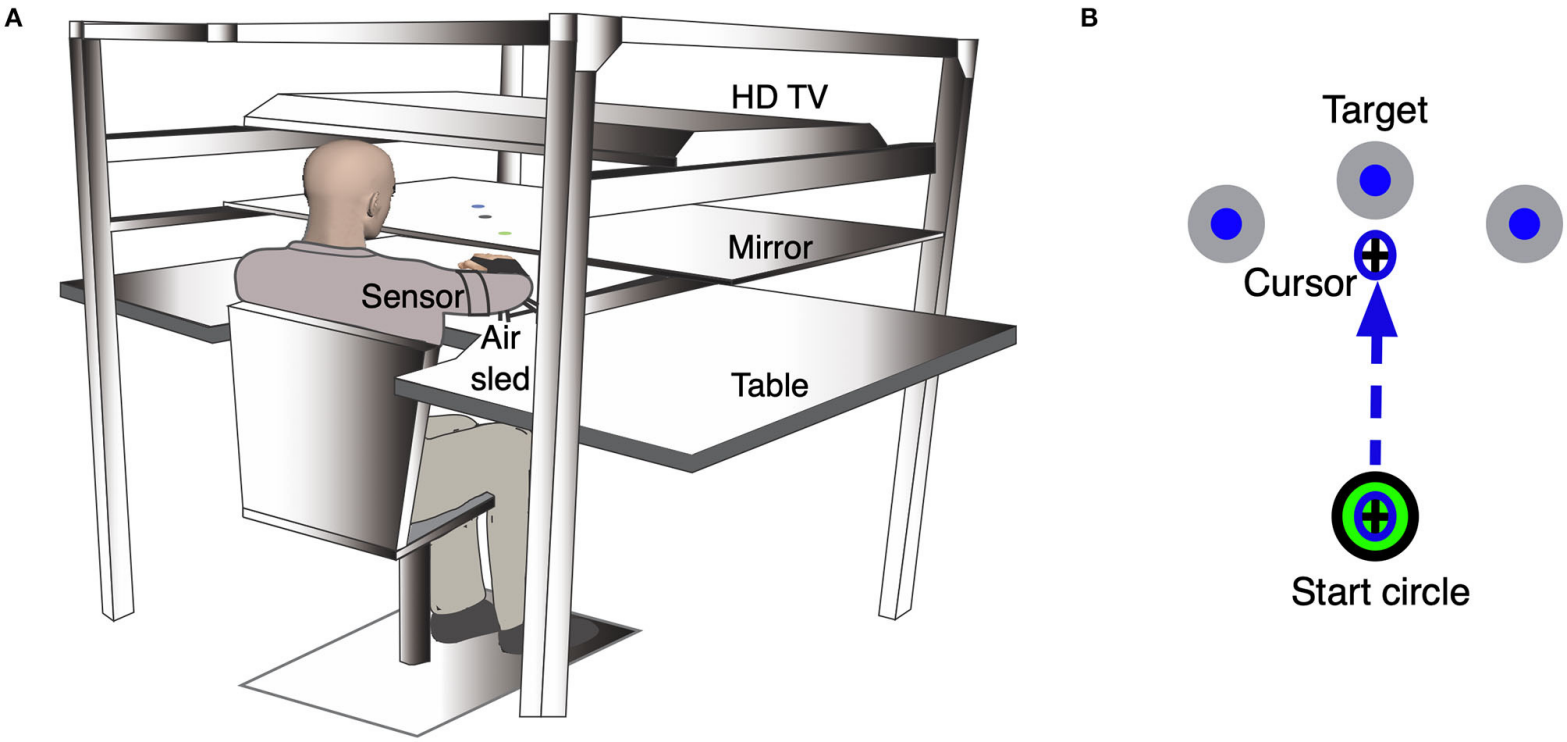
Experimental setup for the kinematic task. **(A)** An illustration of the KineReach setup. Participants are seated in front of a table with the chin resting on a mirrored surface that displayed the task and occluded view of the hands. Sensors were placed on the ipsilesional hand and upper arm. The hand was supported on an air sled. **(B)** Schematic of the ipsilesional arm reaching task showing movement of a cursor to the middle target. Participants were asked to move the cursor (representing the position of the hand) quickly into a target that appeared in one of three directions in a pseudorandom manner. The task consisted of 99 trials.

We used the KineReach virtual reality motion tracking system to record and assess kinematics of ipsilesional arm reaching (see Figure 1A). Participants were seated on a height-adjustable chair, with the chin resting on a horizontal mirrored screen so that the hands were hidden from view. An inverted HD monitor projected the task onto this mirror so that it appeared in the same horizontal plane as the participant's ipsilesional hand. We used an adjustable brace to immobilize all arm joints distal to the elbow. We placed two 6-degree-of-freedom magnetic sensors (trakStar® Ascension Technology) on the upper arm and hand to record limb position and orientation at 116 Hz. We computed arm movements from digitized bony landmarks to estimate finger, wrist, elbow, and shoulder joint positions, as well as shoulder and elbow angles. The participant's hand was supported on an air sled that glided on the horizontal surface in order to reduce the mechanical effects of friction and gravity on movement.

Participants completed a reaching task (99 trials) with their ipsilesional arm (Figure 1B). We asked participants to move a cursor, representing the position of the hand, from a start circle (2 cm diameter) to the target circle (3.5 cm diameter) that appeared 17 cm away, within a two-second window, using a single rapid movement. The target would appear in one of three locations (vertical: 90° from start, outer: 45° clockwise from start, inner: 45° counterclockwise from start) in a pseudorandom order, when testing the ipsilesional right hand. The same targets were presented for participants whose ipsilesional hand is the left, but the counterpart to the left hand's outer target would be the right hand's inner target, and vice versa for the inner target. We provided visual feedback of the cursor continuously for the first 30 trials to familiarize participants with the task. Following these familiarization trials, we removed cursor feedback when the cursor moved out of the start circle. Feedback of the movement hand path was provided at the end of each trial. The task was self-paced.

### Data Analysis

We used custom programs designed in IgorPro (version 6.37; WaveMetrics) to process and analyze all kinematic data. We defined movement onset as the first minimum of tangential velocity that was less than 8% of peak velocity, and movement offset as the first minimum of tangential velocity appearing after peak velocity that was less than 8% of peak velocity. We determined three measures of hand movement performance—deviation from linearity, constant final position error, and variable error, which was measured at peak velocity and at movement end. Deviation from linearity was determined as the hand path's minor axis divided by its major axis, where the major axis was the largest distance between any two points on the path, and the minor axis was the largest distance, perpendicular to the major axis, between any two points (Sainburg et al., 1993; Sainburg, 2002). Higher deviation from linearity signifies more curved trajectories. We calculated final position error as the distance between target center and cursor position at the end of the trial. Higher final position errors signified poor control of postural stabilization mechanisms. Variable error, a measure of consistency across trials, was determined as the distance from cursor position (at either peak velocity or at movement end) to the mean cursor position, and was calculated within subject and target (Schaefer et al., 2009b). We computed the ratio of variable error at final position relative to variable error at peak velocity for each participant, where a ratio >1 signified higher variable error at the end of movement, as compared to the value at peak velocity, and vice versa for a ratio <1. We calculated this measure at each target in each group to determine whether there was an effect of movement direction (Gordon et al., 1994).

We used JMP Pro (Version 14, SAS Institute) to conduct all statistical analyses. We used a one-way ANOVA to determine group (LHD, RHD) differences in each of the following: ipsilesional arm grip strength, UEFM, JTHFT, BI. To test hypothesis one, we used a mixed model ANOVA with lesioned hemisphere (left, right) as the between-subjects variable and target (inner, outer, vertical) as the within-subject variable to test effects on the ratio of variable error. We modeled subject as a random effect. To test hypotheses 2 and 3, we performed linear correlations. We used Pearson's *r* (two-tailed significance) to assess the linear relationship between functional independence (the BI score) and each of the two kinematic measures and three clinical measures of motor performance. We also used Pearson's *r* (two-tailed significance) to assess the linear relationship between each of the two kinematic measures and ipsilesional arm motor performance (JTHFT score). We used a Type I error rate of 0.05.

## Results

### Participant Characteristics

Detailed participant demographics and clinical test scores are presented in Table 1. We used a one-way ANOVA to determine whether LHD (*n* = 10) and RHD (*n* = 10) participants differed with respect to each of the kinematic, clinical and functional independence measures. We found no statistically significant group differences with respect to the contralesional arm clinical measure: UEFM [*F*_(1, 18)_ = 0.54, *p* = 0.47], one of the ipsilesional arm clinical measures: grip strength [*F*_(1, 18)_ = 0.013, *p* = 0.91], or the measure of functional independence: BI [*F*_(1, 18)_ = 0.047, *p* = 0.83]. We found that the JTHFT (ipsilesional arm clinical measure) total score was significantly higher in the LHD group than in the RHD group [*F*_(1, 18)_ = 5.98, *p* = 0.025], suggestive of more extensive ipsilesional arm deficits in LHD individuals, which is consistent with recent findings. However, upon closer examination, we found a statistically significant group difference for the writing component of the JTHFT, but no statistically significant group differences when this writing component was removed from the JTHFT total score [*F*_(1, 18)_ = 1.23, *p* = 0.28]. Because performance on the writing component is related to premorbid writing experience, we removed this component from further analysis, and we have excluded the writing component here on when referring to the JTHFT score.

**Table 1:** Summary of participant information.

**Subject**	**UEFM (/66)**	**JTHFT (s)**	**Ipsilesional grip strength (kg)**	**BI (/100)**	**Type of stroke**
RHD1	9	62.23	33.7	65	Ischemic
RHD2	16	84.99	48.17	90	Ischemic
RHD3	10	101.4	24.7	75	Ischemic
RHD4	6	53.37	18	40	Ischemic
RHD5	17	98.76	14	90	Ischemic
RHD6	10	72.55	32.33	95	Ischemic
RHD7	13	50.82	21.67	80	Hemorrhagic
RHD8	16	69.7	24	80	Hemorrhagic
RHD9	14	42.63	45	85	Ischemic
RHD10	9	44.92	34	85	Ischemic
LHD1	15	154.97	39.7	85	Hemorrhagic
LHD2	18	74.45	34	95	Ischemic
LHD3	6	132.47	32.3	95	Ischemic
LHD4	8	153.15	20	65	Ischemic
LHD5	10	65.65	13.67	75	Hemorrhagic
LHD6	4	349.27	17.42	25	Ischemic
LHD7	14	80.15	38	95	Ischemic
LHD8	10	97.66	31.33	90	Ischemic
LHD9	10	115.98	29.33	45	Hemorrhagic
LHD10	12	111.96	34.67	95	Ischemic

*Scores for the upper extremity portion of the Fugl-Meyer assessment (UEFM), Jebsen-Taylor Hand Function Test (JTHFT), ipsilesional arm grip strength, the Barthel Index (BI), and type of stroke are listed for each of the 10 stroke survivors (RHD: right hemisphere damage; LHD: left hemisphere damage)*.

### Ipsilesional Arm Performance Is Correlated With Functional Independence in the LHD Group, While Contralesional Arm Impairment Is Correlated With Functional Independence in the RHD Group

**Figure 2:**
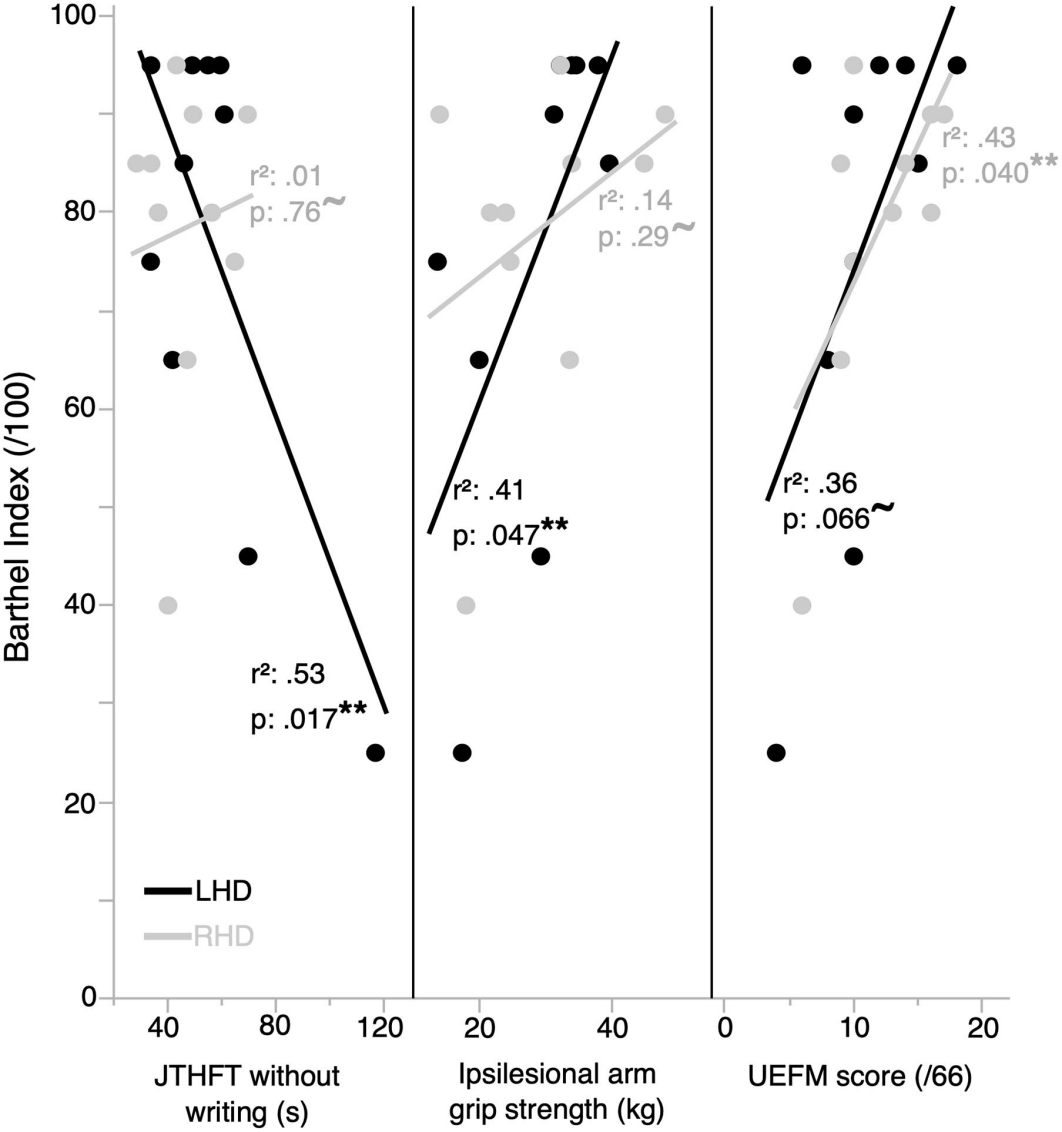
Functional independence is correlated with clinical measures of performance in the ipsilesional and contralesional arms. Functional independence is better correlated with ipsilesional arm clinical measures of performance in the LHD group (*n* = 10), and by contralesional arm impairment in the RHD group (*n* = 10). **represents statistically significant correlations; ^**~**^represents correlations that are not statistically significant.

We examined the relationship between functional independence and clinical measures of motor performance in LHD and RHD participants. Figure 2 shows the relationship between functional independence (BI score) and each clinical measure for the ipsilesional arm (JTHFT, grip strength) and the contralesional arm (UEFM). We regressed BI with each of these clinical measures, as reflected by Pearson's r^2^, which is shown on each panel of Figure 2. The LHD group exhibited a statistically significant linear relation between each of BI and JTHFT [*r*_(10)_ = −0.73, *p* = 0.017], and BI and grip strength [*r*_(10)_ = 0.64, *p* = 0.047]. There was a statistically nonsignificant linear relation between BI and the UEFM [*r*_(10)_ = 0.60, *p* = 0.066]. In contrast, the RHD group showed a statistically significant linear relation between BI and the UEFM [*r*_(10)_ = 0.66, *p* = 0.040], and a statistically nonsignificant linear relation between each of BI and the JTHFT [*r*_(10)_ = 0.11, *p* = 0.76], and BI and grip strength [*r*_(10)_ = 0.37, *p* = 0.29].

### Ipsilesional Arm Kinematics

**Figure 3:**
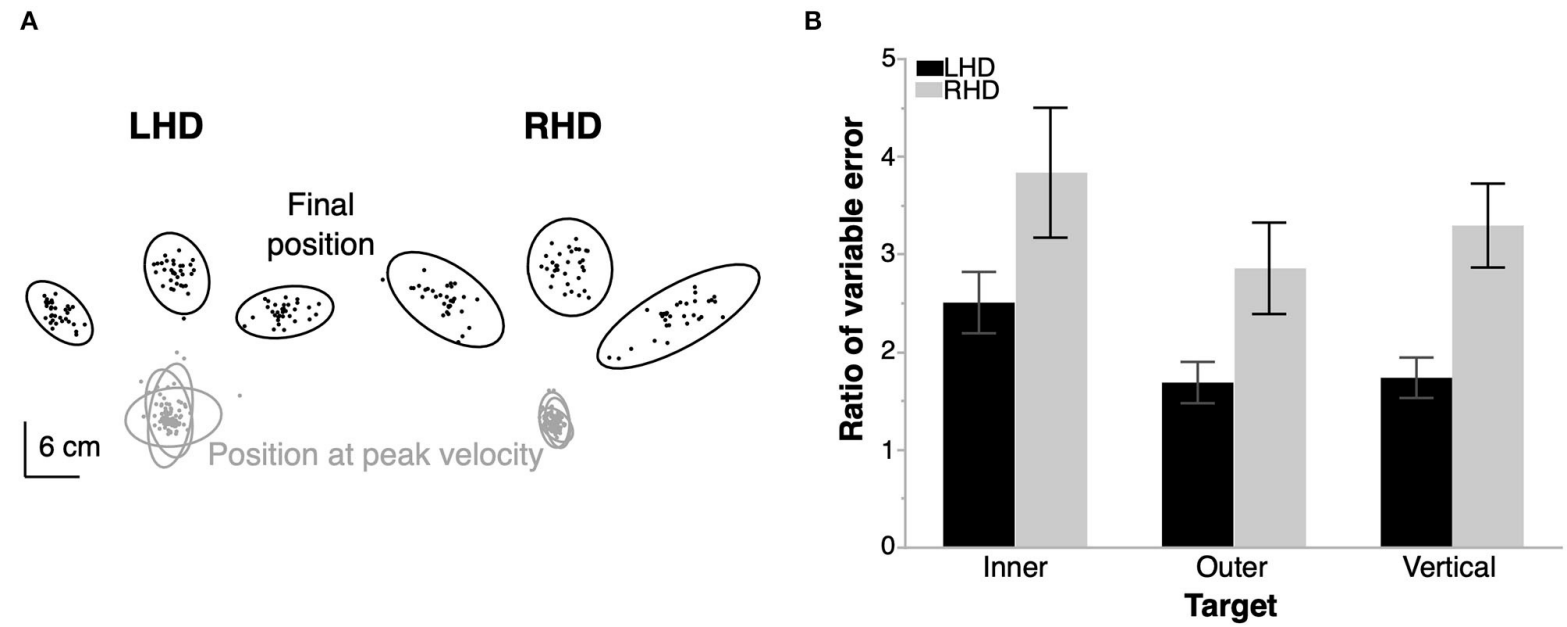
Ipsilesional arm reaching task. **(A)** Location of the hand at peak velocity and at the end of movement for each trial in a representative left- (LHD) and right- (RHD) hemisphere damaged individual. Ellipses represent the 99% confidence interval of the data from each target for each individual. **(B)** Mean ratio of variable error at end of movement relative to that at peak velocity for each target in a group. RHD produces a statistically significantly higher ratio of variable error than LHD. Error bars represent 1 s.e.m. across participants in each group (10 LHD, 10 RHD).

In order to better understand the different dependencies of functional independence on ipsilesional arm function for our two groups, we assessed ipsilesional arm kinematics. Figure 3A shows the variance in two critical hand-path points, position at peak velocity and position at the end of movement, for a representative LHD and RHD individual. We observed that hand paths in both groups were generally more variable at the end of movement than during the initial segment of the trajectory; however, RHD individuals showed higher variability at the end of movement than did LHD individuals. Figure 3B shows the mean ratio of variable error at end of movement to variable error during movement for each group and target. The plot reveals values greater than 1 for all conditions, which signifies higher variable error at end of movement than during movement, regardless of group and target. However, the RHD group had a significantly greater ratio of variable error than did the LHD group [mixed model ANOVA, *F*_(1, 18)_ = 8.7, *p* = 0.0086, 95% CI [−1.16, −0.19]]. There was also a statistically significant main effect of target [*F*_(2, 36)_ = 4.3, *p* = 0.021], with a higher ratio for the inner target compared to the vertical target [*p* = 0.0075, 95% CI [0.145, 0.89]] and lower ratio for the outer target compared to the vertical target [*p* = 0.045, 95% CI [−0.75, −0.0097]]; however, the group x target interaction effect was not statistically significant [*F*_(2, 36)_ = 0.19, *p* = 0.83].

### Ipsilesional Arm Kinematics Are Correlated With Functional Independence in LHD but Not RHD Individuals

**Figure 4:**
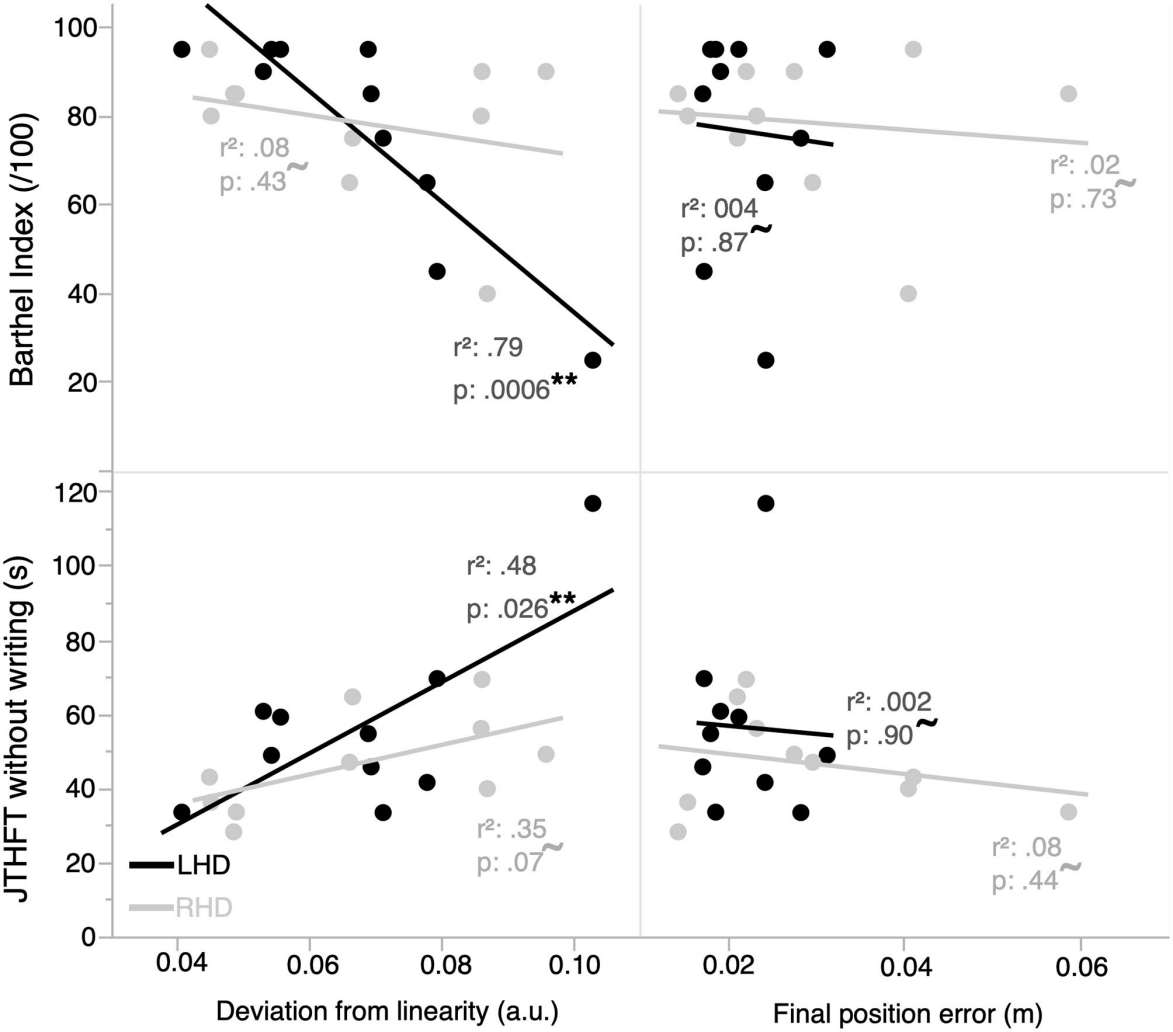
Functional independence and ipsilesional arm motor performance are correlated with kinematic measures of performance in the ipsilesional arm. Functional independence and motor performance in the LHD group (*n* = 10) are better correlated with an ipsilesional arm optimal trajectory control vs. impedance control measure of hand kinematic performance. The RHD group (*n* = 10) does not have a statistically significant linear relation between any of the kinematic measures and functional independence/ipsilesional motor performance. **represents statistically significant correlations; ^**~**^represents correlations that are not statistically significant.

Figure 4 shows the two kinematic performance measures, which evaluated optimal trajectory control and impedance control, plotted by group with respect to functional independence (BI score) and ipsilesional arm motor performance (JTHFT). Performance in LHD individuals showed a statistically significant linear relation between BI and deviation from linearity [*r*_(10)_ = −0.89, *p* = 0.0006], and a statistically nonsignificant linear relation between BI and final position error [*r*_(10)_ = −0.059, *p* = 0.87]. Similarly, there was a statistically significant linear relation between JTHFT and deviation from linearity [*r*_(10)_ = 0.69, *p* = 0.026], and a statistically nonsignificant linear relation between JTHFT and final position error [*r*_(10)_ = −0.045, *p* = 0.90].

In contrast, performance in RHD individuals did not exhibit a statistically significant linear relation between each of BI and deviation from linearity [*r*_(10)_ = −0.28, *p* = 0.43], BI and final position error [*r*_(10)_ = −0.13, *p* = 0.73], JTHFT and deviation from linearity [*r*_(10)_ = 0.59, *p* = 0.070], and JTHFT and final position error [*r*_(10)_ = −0.28, *p* = 0.44].

### The Role of Apraxia on the Relationship Between Functional Independence and Ipsilesional Arm Motor Deficits

Previous work has indicated that apraxia potentiates ipsilesional arm motor deficits, specifically in LHD individuals (Mutha et al., 2010; Maenza et al., 2020). We used the 15-item apraxia battery (Haaland and Flaherty, 1984) to identify the presence of apraxia. We identified five of the ten individuals in the LHD group as apraxic, indicated as a score of 11 or less on the apraxia assessment that scores errors in hand or arm orientation, shape of the hand, position errors, and body-part-as-object errors. No individuals in the RHD group were identified as apraxic. We then categorized the LHD group as apraxic or non-apraxic to determine whether apraxia affects the amplitude of deficits and functional independence. We found no statistically significant differences between these subgroups on any of the clinical or kinematic measures described above. When re-examining the linear relationships between functional independence and ipsilesional arm clinical and kinematic measures in each of the apraxic and non-apraxic groups, we found that the apraxic group exhibited a statistically significant linear relationship between BI and deviation from linearity [*r*_(5)_ = −0.99, *p* = 0.0019]. No other linear relationships were statistically significant. However, these analyses were conducted on a small sample (*n* = 5), and therefore cannot provide a definitive conclusion regarding the role of apraxia on the relationship between functional independence and ipsilesional arm motor deficits.

## Discussion

We examined upper arm function in severely paretic chronic stroke survivors to determine hemisphere-dependent kinematic and clinical correlates of functional independence. Our predictions were based on a model of lateralized motor control, which states that the left hemisphere mediates predictive control of trajectory dynamics and the right hemisphere mediates impedance control in order to achieve accurate and stable final positions (Sainburg, 2002). Our reaching task results showed that both RHD and LHD stroke survivors produced more variable movements at the end of the trajectory than during its initial segment, with RHD participants producing more variable movements than LHD participants. Most surprising was our finding that in the RHD group, contralesional not ipsilesional arm impairment was better correlated with functional independence, unlike in the LHD group. Our findings seem at odds with a recent study conducted with right-hand dominant stroke survivors, which found that LHD individuals used their contralesional arm more than did RHD individuals (Yadav et al., 2019). Our current findings suggest that even though LHD survivors may use the contralesional arm more than RHD survivors (Yadav et al., 2019), impairments in the contralesional arm are less likely to correlate with functional independence limitations for LHD survivors. The greater use of the contralesional arm in LHD than RHD might reflect a handedness component in driving paretic arm use post stroke, at least at lower impairment levels. The greater use of the contralesional arm in LHD is probably associated with bilateral assistance activities during activities of daily living, which primarily rely on the manipulative capabilities of the ipsilesional, less-effected, arm. It is plausible that this might explain the finding that functional independence depends to a greater extent on functioning of the ipsilesional than the contralesional arm in this group.

Our kinematic results from the ipsilesional arm reaching task are supported by findings from previous work in stroke survivors with a larger range of ipsilesional impairment (Schaefer et al., 2009a,b). In general, we observed greater variability in hand path trajectories at the end of movement in the RHD group than in the LHD group. Both groups produced more variable hand paths at the end of movement than during the initial segment of the trajectory, as shown by variable error ratios that were greater than one regardless of target location and lesioned hemisphere. We found that initial trajectory deficits were better correlated with functional independence in the LHD group than the RHD group; however, final position deficits did not correlate well with functional independence in the RHD group. There was a positive linear relationship between trajectory control and functional performance in LHD individuals, but there was no evidence of a relationship between impedance control and functional performance in RHD individuals. Previous work that examined the relationship between movement variables and ipsilesional arm functional performance found that reaction time, which is a reflection of movement preparation involving cognitive and little to no kinematic aspects of control and coordination, was strongly correlated with functional performance in the RHD group, but not in the LHD group (Schaefer et al., 2009b). Hand path curvature, which is a measure of trajectory control, was strongly correlated with functional performance in the LHD group, but not in the RHD group. Our findings are consistent with these results, and suggests that another set of factors play a major role in defining functional outcomes in RHD individuals with severe paresis.

The lack of correlation between ipsilesional arm motor-related variables and functional independence in RHD individuals is perplexing given the reduced levels of movement produced with the paretic arm compared to the less-affected arm. In both groups, the severity of paresis indicates that these stroke survivors were not able to functionally grasp, release, and transport objects with the affected arm (Lang et al., 2007; Michielsen et al., 2012), placing a large functional burden on the less-affected arm. We examined whether apraxia may have influenced our findings since previous work has indicated that apraxia potentiates ipsilesional arm motor deficits (Mutha et al., 2010). Our sample had five individuals with apraxia, with all five being LHD individuals. Thus, we find it unlikely that apraxia can explain our findings of a poor relationship between ipsilesional arm motor-related deficits and functional independence in the RHD group. We also found that removing the apraxic individuals from the LHD group largely maintained the linear relationships between functional independence and clinical/kinematic measures of ipsilesional arm motor performance seen in the non-apraxic group. While apraxia represents a potential confound, it was not a factor in our study design, and the limited number of apraxic individuals in our sample precludes a definitive conclusion.

We now suggest that lateralized cognitive-motor factors might influence the relationship between ipsilesional motor deficit and functional independence in RHD individuals. Although studies have found that the majority of variance in upper extremity function can be explained by the severity of paresis (Lang et al., 2013), other studies have shown that cognitive deficits, such as attention and visual memory play a significant role in the functional recovery (Ramsey et al., 2017). Previous studies that examined the lateralization of cognitive-motor processes, such as working memory, visuospatial orientation, and sequencing, found that each hemisphere contributes to certain processes more than others (Barthelemy and Boulinguez, 2001; Hanna-Pladdy et al., 2001; Kessels et al., 2002; Philipose et al., 2007). The hemisphere-dependent contributions of cognitive-motor processes in achieving functional independence are still unclear, and a deeper examination of these may aid in determining the surprising results produced by the RHD group. It must be noted, though, that even the LHD group showed a moderate correlation between BI and UEFM, indicating (1) that even in this severely paretic group, the contralesional arm may contribute some assistance to activities of daily living, and/or (2) other factors that might vary with severity of contralesional deficits, such as cognitive motor processes, may contribute to limitations in functional independence in both groups of stroke survivors. Thus, we suggest that future research should examine the impact of factors other than motor performance in achieving functional independence. Unfortunately, this was beyond the scope of this study.

Among the limitations of our study, we acknowledge the small sample size and variable lesion size and location in our participant group. This was a convenience sample that is related to our rigorous exclusion criteria, which only allowed recruitment of participants with UEFM scores that were <20. We perceive benefits in studying populations that fall within published ranges of impairment due to stroke (Woytowicz et al., 2017) in order to provide a better understanding of the challenges faced by chronic stroke survivors. Another limitation that can be addressed in the future is the measure of functional independence—the Barthel Index is a 10-item questionnaire that provides an omnibus score without any task-level resolution, and can produce ceiling effects as seen in our dataset. It may be meaningful to use additional clinical assessments of upper arm function that are more fine-grained, such as the Motor Activity Log and the Manual Ability Measure. All clinical measures of functional independence present range effects that could influence our findings. For example, 65% of our participants had a score of 60 (out of 100) or higher on the Barthel Index, which is suggestive of moderate-to-slight dependency (Shah et al., 1989). It is important in future work to examine stroke survivors with a broader range of functional independence values in order to determine whether specific ranges have stronger influences on the relationships with ipsilesional motor deficits than others. The lack of a detailed cognitive battery is another limitation which may have influenced our interpretation of the results. In addition to using clinical measures of functional independence, we may examine upper extremity use for an extended period in daily life via continuous data derived from accelerometry (Lang et al., 2013).

The present study sought to determine whether kinematic and clinical measures of motor performance and impairment in each arm of severely paretic stroke survivors differentially correlated with functional independence in LHD and RHD individuals. To our knowledge, this is the first study to address this question in severely paretic stroke survivors. We found that both kinematic and clinical measures (JTHFT and grip strength) of ipsilesional motor performance were linearly related to functional independence (BI) in LHD, but not RHD individuals. In addition, we found that our measure of contralesional impairment (UEFM) was only linearly related with functional independence in the RHD group. Previous reports used a similar kinematic task and/or clinical measures in patients with mild-to-moderate paresis, and reported a dependence of functional performance measures on ipsilesional arm function (Schaefer et al., 2009b; Varghese and Winstein, 2020), which is understandable because of the importance of the ipsilesional arm on function in patients with even mild deficits (Maenza et al., 2020). In addition, in patients with mild-to-moderate paresis, previous reports have indicated the dependence of contralesional hand impairment level on function. In these patients, the contralesional arm participates, often as the lead manipulator in functional activities. However, in our current group of stroke survivors with severe paresis and no voluntary hand control, the contralesional arm cannot participate in functional manipulations. Therefore, we found these findings in the RHD group somewhat perplexing because differences in impairment among participants in this group with very severe paresis would not be expected to have a large influence on functional independence. We now hypothesize that these findings in the RHD group may be related to covariates associated with cognitive and perceptual motor processes, such as working memory and attention, which we did not assess in this study. Based on our findings, it is plausible that rehabilitation focused on improving motor control and coordination in the ipsilesional arm may help improve functional independence in LHD survivors with severe contralesional paresis. However, more research is necessary to determine the factors that are related to functional independence limitations in RHD survivors with severe paresis.

## Data Availability Statement

The raw data supporting the conclusions of this article will be made available by the authors, without undue reservation.

## Ethics Statement

The studies involving human participants were reviewed and approved by Pennsylvania State University Institutional Review Board, University of Southern California Institutional Review Board. The patients/participants provided their written informed consent to participate in this study.

## Author Contributions

SJ performed experiments, analyzed the data, interpreted findings, and wrote the original draft. DG and DW interpreted findings, reviewed, and approved the submitted version. CW conceived the research, interpreted findings, reviewed, and approved the submitted version. RS conceived the research, designed the study, contributed to data analysis, interpreted findings, reviewed, and approved the submitted version. All authors contributed to the article and approved the submitted version.

## Conflict of Interest

The authors declare that the research was conducted in the absence of any commercial or financial relationships that could be construed as a potential conflict of interest.
